# Plaster Impressions

**Published:** 1860-10

**Authors:** 


*Plaster Impressions.*—A writer in the Register for June advocates plaster for impressions in all available cases, as giving superior results, more cleanly and accurate, which we fully endorse. It is often too lightly held in contact with the parts to be taken, and managed with too much impatience, but, properly used, it is infinitely superior to all other materials for this purpose. B.

